# Endogenous CD28 takes the driver’s seat in 4-1BB co-stimulated CAR T cells

**DOI:** 10.1016/j.omton.2026.201192

**Published:** 2026-04-15

**Authors:** Jason H. Tong, Scott H. Olejniczak

**Affiliations:** 1 Department of Immunology, Roswell Park Comprehensive Cancer Center, Buffalo, NY 14263, USA

## Main text

**Figure 1:**
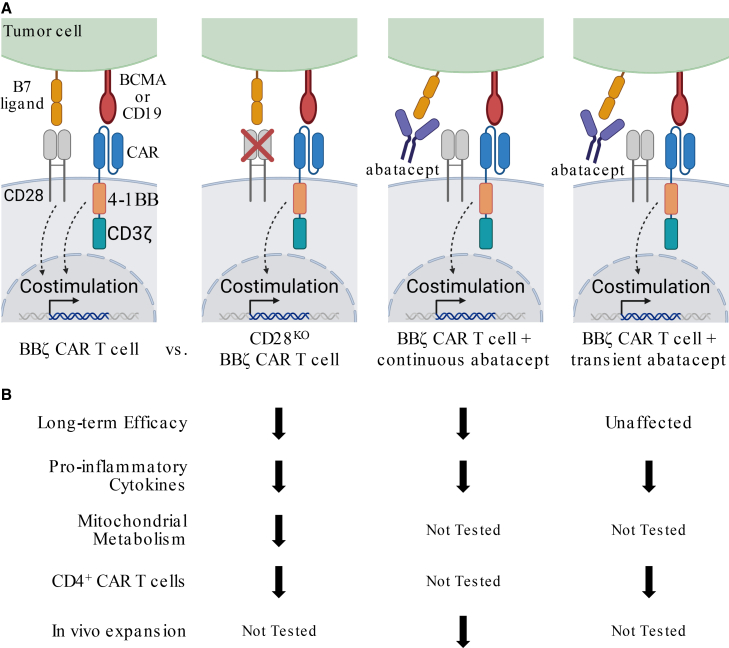
Schematic of receptor-ligand interactions and effects of CD28 modulation on CAR T cell function described in Lieberman et al. (A) Parallel co-stimulatory signals originating from endogenous CD28 and the CAR 4-1BB signaling motif upon BBζ CAR T cell interaction with target cells expressing B7 ligands are disrupted by CD28 knockout (CD28^KO^) or temporally blocked by continuous (intraperitoneal injection 3× per week over the entire course of experiments) versus transient (intraperitoneal injection on days −1, 1, 3, 5, and 7 relative to CAR T cell injection) abatacept exposure. (B) Consequences of the disruption of endogenous CD28 signaling in BBζ CAR T cells via CD28^KO^, continuous abatacept exposure, or transient abatacept exposure for CAR T cell efficacy, metabolism, expansion, and TME cytokine release. (A, Created with BioRender.com).

Inhibiting the pro-survival CD28 signal on multiple myeloma (MM) cells by blocking its interaction with B7 proteins, CD86 and CD80, can resensitize refractory MM to chemotherapy.^1^ In our recent article published in *Blood Cancer Discovery*,^2^ we sought to extend these findings to chimeric antigen receptor (CAR) T cell therapy using mouse models of MM. Contrary to expectations, CD28 blockade limited persistent tumor control by 4-1BB co-stimulated (BBζ) CAR T cells, revealing a novel role for endogenous CD28 in supporting CAR T cell efficacy (Figure 1).

CAR T cell therapy has revolutionized the treatment of patients with relapsed/refractory hematological malignancies, including MM and B cell leukemia/lymphoma (BCL). Five of the seven FDA-approved CARs on the market, including both used in MM, incorporate a 4-1BB signaling motif in the intracellular CAR signaling domain. In our study,^2^ abatacept, a cytotoxic T-lymphocyte protein 4 - immunoglubulin (CTLA-4-Ig) fusion protein used to treat autoimmune diseases, was initially employed to block CD28:B7 interactions in mouse and human xenograft MM models, with the expectation that B cell maturation antigen (BCMA)-targeted, BBζ CAR T cells would function normally in the absence of endogenous CD28 signaling, while MM cells would be sensitized to CAR T killing. Abatacept did not impact CAR T cell cytotoxicity or cytokine production *in vitro*, yet injection of abatacept three times per week over the course of *in vivo* experiments, referred to as “continuous abatacept,” significantly decreased persistent *in vivo* CAR T cell anti-myeloma activity. These findings extend beyond MM, as continuous abatacept similarly reduced the persistent antitumor activity of CD19-targeted, BBζ CAR T cells in A20 BCL-bearing BALB/c mice.

Abatacept blocking experiments, along with prior publications hinting at the importance of CD28 signaling in BBζ CAR T cell responses,^3^^,^^4^^,^^5^^,^^6^^,^^7^ raised the intriguing possibility that signaling through endogenous CD28 may contribute to the persistent antitumor activity of BBζ CAR T cells. Using a tamoxifen-inducible CD28 knockout mouse and CRISPR-Cas9-mediated CD28 knockout in human CAR T cells, we demonstrated that endogenous CD28 supports CAR T cytotoxicity over the course of repeated *in vitro* stimulations with CD86-expressing, but not CD86-deficient, MM cells, and extends the survival of CAR T cell-treated MM-bearing mice. Moreover, CD28 knockout shortened B cell aplasia following injection of murine CD19-targeted BBζ CAR T cells into C57BL/6 mice. Together, these data from complementary mouse models provided compelling evidence that signaling through endogenous CD28 expressed on BBζ CAR T cells supports their persistent *in vivo* activity.

Mechanistically, we found that known downstream functions of CD28 signaling are conserved in BBζ CAR T cells, likely contributing to persistent antitumor function. In agreement with prior work showing that CD28 co-stimulation reprograms mitochondrial metabolism to sustain T cell memory,^8^ CD28 knockout BBζ CAR T cells exhibited impaired mitochondrial respiration, a lower NADH:NAD^+^ ratio, and elevated mitochondrial reactive oxygen species (mROS) (Figure 1). *In vivo,* the frequency and number of CD4^+^ CAR T cells in bone marrow isolated from femurs of MM-bearing mice was reduced by CD28 blockade or knockout, yet elimination of MM burden was similar 1 week following CAR T injection. These data are reminiscent of the initial clinical response to CAR T therapy in most patients followed by less frequent extended remissions characterized by CD4^+^ BBζ CAR T cells exhibiting high CD28 expression^9^ and suggest that CD28 may be a key factor in CD4^+^ BBζ CAR T cell persistence.

Clinically, we observed that expression of the CD28 ligand CD86 was common on MM cells in a small cohort of CAR T cell-treated patients with MM (*n* = 23). CD86 expression was associated with a much higher frequency of grade 2 or greater cytokine release syndrome (CRS) and use of steroids to treat immune-related adverse events (irAEs), although, due to the small number of patients, multivariate testing revealed no statistically significant differences. In agreement, a recent quantitative multiplex co-immunoprecipitation study found that CD28 signaling complexes in CD19-targeted BBζ CAR T cells correlated with and could predict CRS in pediatric and young adult patients with B-cell acute lymphoblastic leukemia (B-ALL).^7^ In line with these clinical observations, we found that CD28 knockout and/or blockade decreased pro-inflammatory cytokine release in the bone marrow tumor microenvironment (TME) of MM- or BCL-bearing mice. As expected, no overt toxicity was observed in CAR T cell-treated mice, potentially because elevated pro-inflammatory cytokines were confined to the TME. Affected cytokines were mostly myeloid-derived and differed between MM and BCL models, suggesting that CD28 interaction with B7 proteins expressed on myeloid cells in the TME may contribute to the cytokine cascade underlying CRS and other CAR T therapy-associated irAEs.

Because the effects of abatacept on cytokine release occurred within a week of CAR T cell injection, but its effect on tumor control were not observed until several weeks later, we next tested whether transient CD28 blockade could dampen cytokine production without affecting CAR T cell antitumor efficacy. Xenograft studies in human MM and murine BCL models demonstrated that transient 7-day treatment with abatacept did not impact the survival of BBζ CAR T cell-treated mice, while, at the same time, inflammatory cytokine levels in the TME were reduced. This finding raised the prospect that transient CD28 blockade could be a novel approach to limiting irAEs in CAR T cell-treated patients.

When considering co-stimulation in CAR T cells, most studies focus on modulating the co-stimulatory domain within second- or third-generation CARs to promote favorable T cell properties.^10^ Only recently has the importance of endogenous co-stimulation been appreciated. Single-cell transcriptomics examining links between clinical response in patients with B-ALL and properties of infused CD19-targeted BBζ CAR T cell products identified CD28 and CD28 downstream Th2 gene signatures as significantly associated with durable remission.^5^ Similarly, patients with decade-long remissions following BBζ CAR T cell therapy contained circulating CD4^+^ BBζ CAR T cells exhibiting high CD28 expression that were viewed as critical for maintaining durable tumor control.^9^ As discussed above, Draper et al.^7^ further showed that the endogenous CD28 interactome is associated with CAR T therapy-associated CRS. Preclinical studies further implicated endogenous CD28 signaling in regulating BBζ CAR T cell activity by showing that CD80 co-expression^4^ or CTLA-4 knockout^3^ improved tumor control by BBζ CAR T cells and that a lack of co-stimulatory ligands on chronic lymphocytic leukemia (CLL) cells reduced the antitumor activity of BBζ CAR T cells.^6^

While these clinical and preclinical studies implied that endogenous CD28 promotes BBζ CAR T cell antitumor activity, our study provides the first direct evidence that endogenous CD28 does, in fact, promote durable tumor control by BBζ CAR T cells. Additional evidence supporting the role of endogenous CD28 in promoting irAEs associated with CAR T cell therapy and the demonstrated ability to block CD28-induced pro-inflammatory cytokine release during the initial CAR T cell antitumor response without affecting long-term survival add to the potential clinical impact. In summary, accumulating evidence supports modulation of CAR signaling crosstalk with endogenous receptors as a strategy to improve CAR T cell therapy outcomes. Future studies aimed at understanding how this crosstalk occurs will bring us closer to a signaling “Goldilocks” point at which CAR T cells optimally respond to the many signals they encounter to yield durable antitumor responses.
